# [*N*′-(3-Meth­oxy-2-oxidobenzyl­idene)nicotinohydrazidato]dimethyl­tin(IV)

**DOI:** 10.1107/S160053680903726X

**Published:** 2009-09-19

**Authors:** Zhongjun Gao, Wandong Chen, Yanbao Wang, Guangwang Zhao

**Affiliations:** a Department of Chemistry and Chemical Engineering, Jining University, Shandong 273155, People’s Republic of China

## Abstract

In the title complex, [Sn(CH_3_)_2_(C_14_H_11_N_3_O_3_)], the Sn atom is in a distorted trigonal-bipyramidal coordination, with Sn—O distances of 2.138 (2) and 2.176 (2) Å. The dihedral angles between the two chelated benzene rings and the O—Sn—N group are 71.73 (9) and 83.30 (9)°.

## Related literature

For covalent radii, see: Sanderson (1967 ▶). For bond-lengh data, see: Yang *et al.* (1999 ▶). For a related structure, see: Yearwood *et al.* (2002 ▶).
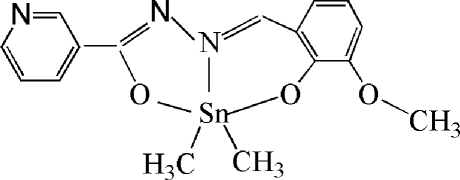


## Experimental

### 

#### Crystal data

[Sn(CH_3_)_2_(C_14_H_11_N_3_O_3_)]
*M* *_r_* = 418.02
Monoclinic, 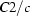

*a* = 26.121 (8) Å
*b* = 9.795 (3) Å
*c* = 13.227 (4) Å
β = 103.666 (4)°
*V* = 3288.4 (17) Å^3^
*Z* = 8
Mo *K*α radiation
μ = 1.57 mm^−1^
*T* = 298 K
0.35 × 0.16 × 0.12 mm

#### Data collection

Bruker SMART CCD area-detector diffractometer
Absorption correction: multi-scan (*SADABS*; Sheldrick, 1996 ▶) *T*_min_ = 0.609, *T*_max_ = 0.834
9367 measured reflections
3574 independent reflections
3115 reflections with *I* > 2σ(*I*)
*R*_int_ = 0.023

#### Refinement

*R*[*F* ^2^ > 2σ(*F* ^2^)] = 0.032
*wR*(*F* ^2^) = 0.087
*S* = 1.00
3574 reflections
211 parameters
H-atom parameters constrained
Δρ_max_ = 0.48 e Å^−3^
Δρ_min_ = −0.54 e Å^−3^

### 

Data collection: *SMART* (Siemens, 1996 ▶); cell refinement: *SAINT* (Siemens, 1996 ▶); data reduction: *SAINT*; program(s) used to solve structure: *SHELXS97* (Sheldrick, 2008 ▶); program(s) used to refine structure: *SHELXL97* (Sheldrick, 2008 ▶); molecular graphics: *SHELXTL* (Sheldrick, 2008 ▶); software used to prepare material for publication: *SHELXTL*.

## Supplementary Material

Crystal structure: contains datablocks I, global. DOI: 10.1107/S160053680903726X/kp2231sup1.cif

Structure factors: contains datablocks I. DOI: 10.1107/S160053680903726X/kp2231Isup2.hkl

Additional supplementary materials:  crystallographic information; 3D view; checkCIF report

## Figures and Tables

**Table 1:** Selected bond lengths (Å)

C15—Sn1	2.092 (3)
C16—Sn1	2.098 (3)
N2—Sn1	2.200 (3)
O2—Sn1	2.138 (2)
O3—Sn1	2.176 (2)

**Fig. 1.**
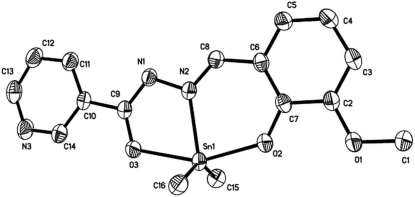
The molecular structure of the title complex with 30% displacement ellipsoids (H atoms omitted for clarity).

## supplementary crystallographic information

### Comment

In the structure of the title compound, (I), (Fig. 1) the Sn1 atom is in a distored trigonal-bipyramidal coordination formed by a tridentate ligand of the azomethine N atom, the hydroxyl O atom and the carbonyl O atom (Table 1). The dihedral angles between the two chelated benzene rings: O2—Sn1—N2 and O3—Sn1—N2 are 83.30 (9) and 71.73 (9)°, respectively. The Sn atom is coordinated in distorted trigonal-bipyramidal arrangement with O2 and O3 located in the axial positions [O2—Sn1—O3 = 155.02 (9)°] and the C15, C16 and N2 in the equatorial positions. The sum of the C15—Sn1—C16 [146.08 (14)], C15—Sn1—N2 [106.13 (11)] and C16—Sn1—N2 [107.23 (11)°] bond angles is 359.44°, indicating approximate co-planarity for these atoms. Comparing the length of CN 1.270Å and C—N 1.470 Å, both bonds, C8—N2 (1.287 (4) Å) and C9—N1 (1.304 (4) Å) should be CN. The N1—N2 bond (1.400 (3) Å) is a single-bond (Yang *et al.*, 1999). The Sn1—N2 distance is close to the sum of the covalent radii of 2.15Å (Sanderson, 1967), indicating a strong Sn—N interaction. Very similar structural parameters were observed in the compound studied by Yearwood *et al.* (2002).

### Experimental

A mixture of dimethyltin oxide (0.3295 g, 2.0 mmol) and 3-methoxy-2-oxideobenzaldehyde(3-pyridyl)methyl-hydrazone (0.5420 g, 2.0 mmol) in methanol (50 ml) was heated under reflux for 6 h. The obtained clear solution was evaporated under vacuum. The product was crystallized from a mixture of dichloromethane/ethanol (1:1) to yield blocks of (I). Yield 0.5936 g, 71%, m.p. 453 K, analysis, calculated for C_16_H_17_N_3_O_3_Sn: C 45.97, H, 4.10; N 10.06%; found: C 46.02, H 4.05, N, 10.09%.

### Refinement

H atoms were positioned with idealized geometry with C—H = 0.93 Å for aromatic H atomds and 0.96 Å for methyl H atoms and were constrained to ride on their parent atoms with *U*_iso_(H) = 1.2*U*_eq_(C) or 1.5*U*_eq_(for methyl groups).

### Crystal data

**Table:**

[Sn(CH_3_)_2_(C_14_H_11_N_3_O_3_)]	*F*(000) = 1664
*M**_r_* = 418.02	*D*_x_ = 1.689 Mg m^−^^3^
Monoclinic, *C*2/*c*	Mo *K*α radiation, λ = 0.71073 Å
Hall symbol: -C 2yc	Cell parameters from 4244 reflections
*a* = 26.121 (8) Å	θ = 2.2–27.6°
*b* = 9.795 (3) Å	µ = 1.57 mm^−^^1^
*c* = 13.227 (4) Å	*T* = 298 K
β = 103.666 (4)°	Block, yellow
*V* = 3288.4 (17) Å^3^	0.35 × 0.16 × 0.12 mm
*Z* = 8	

### Data collection

**Table:**

Bruker SMART CCD area-detector diffractometer	3574 independent reflections
Radiation source: fine-focus sealed tube	3115 reflections with *I* > 2σ(*I*)
graphite	*R*_int_ = 0.023
φ and ω scans	θ_max_ = 27.0°, θ_min_ = 1.6°
Absorption correction: multi-scan (*SADABS*; Sheldrick, 1996)	*h* = −33→20
*T*_min_ = 0.609, *T*_max_ = 0.834	*k* = −12→12
9367 measured reflections	*l* = −13→16

### Refinement

**Table:**

Refinement on *F*^2^	Primary atom site location: structure-invariant direct methods
Least-squares matrix: full	Secondary atom site location: difference Fourier map
*R*[*F*^2^ > 2σ(*F*^2^)] = 0.032	Hydrogen site location: inferred from neighbouring sites
*wR*(*F*^2^) = 0.087	H-atom parameters constrained
*S* = 1.00	*w* = 1/[σ^2^(*F*_o_^2^) + (0.0558*P*)^2^ + 0.5213*P*] where *P* = (*F*_o_^2^ + 2*F*_c_^2^)/3
3574 reflections	(Δ/σ)_max_ < 0.001
211 parameters	Δρ_max_ = 0.48 e Å^−^^3^
0 restraints	Δρ_min_ = −0.54 e Å^−^^3^

### Special details

**Table:**

Geometry. All e.s.d.'s (except the e.s.d. in the dihedral angle between two l.s. planes) are estimated using the full covariance matrix. The cell e.s.d.'s are taken into account individually in the estimation of e.s.d.'s in distances, angles and torsion angles; correlations between e.s.d.'s in cell parameters are only used when they are defined by crystal symmetry. An approximate (isotropic) treatment of cell e.s.d.'s is used for estimating e.s.d.'s involving l.s. planes.
Refinement. Refinement of *F*^2^ against ALL reflections. The weighted *R*-factor *wR* and goodness of fit *S* are based on *F*^2^, conventional *R*-factors *R* are based on *F*, with *F* set to zero for negative *F*^2^. The threshold expression of *F*^2^ > σ(*F*^2^) is used only for calculating *R*-factors(gt) *etc*. and is not relevant to the choice of reflections for refinement. *R*-factors based on *F*^2^ are statistically about twice as large as those based on *F*, and *R*- factors based on ALL data will be even larger.

### Fractional atomic coordinates and isotropic or equivalent isotropic displacement parameters (Å^2^)

**Table:**

	*x*	*y*	*z*	*U*_iso_*/*U*_eq_	
C1	−0.17768 (17)	−0.0742 (5)	−0.0099 (5)	0.112 (2)	
H1A	−0.1913	−0.0622	0.0509	0.169*	
H1B	−0.1729	−0.1697	−0.0208	0.169*	
H1C	−0.2021	−0.0368	−0.0693	0.169*	
C2	−0.12735 (12)	0.1271 (3)	0.0369 (2)	0.0486 (7)	
C3	−0.17073 (12)	0.1957 (3)	0.0522 (3)	0.0543 (8)	
H3	−0.2031	0.1515	0.0391	0.065*	
C4	−0.16700 (14)	0.3308 (3)	0.0870 (3)	0.0598 (9)	
H4	−0.1966	0.3761	0.0973	0.072*	
C5	−0.11962 (12)	0.3952 (3)	0.1057 (3)	0.0530 (7)	
H5	−0.1171	0.4852	0.1286	0.064*	
C6	−0.07437 (12)	0.3282 (3)	0.0910 (2)	0.0450 (7)	
C7	−0.07714 (12)	0.1931 (3)	0.0542 (2)	0.0441 (7)	
C8	−0.02708 (12)	0.4074 (3)	0.1159 (2)	0.0461 (6)	
H8	−0.0301	0.4944	0.1421	0.055*	
C9	0.10299 (11)	0.4315 (3)	0.1225 (2)	0.0453 (7)	
C10	0.14890 (11)	0.5246 (3)	0.1563 (2)	0.0458 (7)	
C11	0.14558 (15)	0.6528 (3)	0.1969 (3)	0.0579 (8)	
H11	0.1133	0.6872	0.2032	0.069*	
C12	0.19100 (16)	0.7297 (4)	0.2282 (3)	0.0701 (10)	
H12	0.1898	0.8172	0.2549	0.084*	
C13	0.23784 (17)	0.6734 (4)	0.2190 (3)	0.0701 (11)	
H13	0.2683	0.7251	0.2416	0.084*	
C14	0.19835 (12)	0.4796 (3)	0.1490 (3)	0.0563 (8)	
H14	0.2006	0.3938	0.1202	0.068*	
C15	0.07460 (13)	0.0606 (3)	0.1813 (2)	0.0564 (8)	
H15A	0.0728	−0.0342	0.1625	0.085*	
H15B	0.0544	0.0760	0.2323	0.085*	
H15C	0.1106	0.0858	0.2099	0.085*	
C16	0.03460 (15)	0.1988 (3)	−0.1120 (3)	0.0562 (8)	
H16A	0.0649	0.2438	−0.1258	0.084*	
H16B	0.0036	0.2518	−0.1405	0.084*	
H16C	0.0310	0.1100	−0.1435	0.084*	
N1	0.05804 (9)	0.4727 (2)	0.1381 (2)	0.0477 (6)	
N2	0.01913 (9)	0.3731 (3)	0.10640 (18)	0.0445 (5)	
N3	0.24269 (11)	0.5509 (3)	0.1802 (3)	0.0683 (8)	
O1	−0.12845 (9)	−0.0056 (3)	0.0043 (2)	0.0690 (7)	
O2	−0.03632 (8)	0.1225 (2)	0.03858 (18)	0.0546 (5)	
O3	0.11123 (10)	0.3162 (2)	0.0820 (2)	0.0604 (6)	
Sn1	0.043848 (7)	0.17888 (2)	0.049245 (14)	0.04388 (10)	

### Atomic displacement parameters (Å^2^)

**Table:**

	*U*^11^	*U*^22^	*U*^33^	*U*^12^	*U*^13^	*U*^23^
C1	0.066 (3)	0.083 (3)	0.202 (6)	−0.031 (2)	0.060 (3)	−0.070 (3)
C2	0.0429 (16)	0.0581 (18)	0.0459 (16)	−0.0086 (14)	0.0129 (12)	−0.0022 (14)
C3	0.0378 (16)	0.072 (2)	0.0542 (19)	−0.0055 (14)	0.0135 (14)	0.0008 (15)
C4	0.0459 (18)	0.069 (2)	0.069 (2)	0.0046 (15)	0.0219 (16)	−0.0008 (16)
C5	0.0518 (18)	0.0535 (18)	0.0568 (19)	0.0003 (14)	0.0190 (14)	0.0026 (15)
C6	0.0419 (16)	0.0540 (17)	0.0397 (15)	−0.0042 (12)	0.0107 (12)	0.0063 (12)
C7	0.0394 (15)	0.0581 (18)	0.0354 (14)	−0.0030 (12)	0.0103 (11)	0.0039 (12)
C8	0.0472 (17)	0.0458 (15)	0.0459 (16)	−0.0028 (13)	0.0120 (12)	0.0027 (13)
C9	0.0451 (16)	0.0492 (16)	0.0418 (15)	−0.0110 (13)	0.0104 (12)	0.0012 (13)
C10	0.0479 (16)	0.0466 (16)	0.0421 (15)	−0.0124 (13)	0.0091 (12)	0.0037 (13)
C11	0.061 (2)	0.0536 (18)	0.061 (2)	−0.0123 (15)	0.0173 (16)	−0.0053 (15)
C12	0.082 (3)	0.0534 (19)	0.076 (3)	−0.023 (2)	0.022 (2)	−0.0120 (19)
C13	0.057 (2)	0.079 (3)	0.071 (3)	−0.0297 (18)	0.0076 (18)	−0.0001 (19)
C14	0.0514 (18)	0.0535 (18)	0.062 (2)	−0.0115 (15)	0.0101 (15)	−0.0008 (16)
C15	0.060 (2)	0.061 (2)	0.0445 (17)	−0.0025 (15)	0.0053 (14)	0.0008 (14)
C16	0.075 (2)	0.0510 (18)	0.0452 (18)	−0.0102 (16)	0.0203 (16)	−0.0036 (14)
N1	0.0450 (14)	0.0442 (13)	0.0535 (15)	−0.0077 (11)	0.0107 (11)	−0.0006 (11)
N2	0.0445 (14)	0.0462 (13)	0.0426 (13)	−0.0091 (11)	0.0100 (10)	−0.0005 (11)
N3	0.0507 (17)	0.0673 (19)	0.085 (2)	−0.0174 (14)	0.0116 (14)	−0.0038 (16)
O1	0.0457 (12)	0.0708 (15)	0.0958 (19)	−0.0155 (11)	0.0277 (12)	−0.0276 (14)
O2	0.0400 (11)	0.0565 (13)	0.0689 (15)	−0.0080 (10)	0.0162 (10)	−0.0079 (11)
O3	0.0509 (14)	0.0569 (14)	0.0793 (18)	−0.0155 (10)	0.0269 (12)	−0.0220 (11)
Sn1	0.04134 (14)	0.04977 (15)	0.04093 (14)	−0.01097 (8)	0.01049 (9)	−0.00053 (8)

### Geometric parameters (Å, °)

**Table:**

C1—O1	1.423 (4)	C10—C14	1.390 (4)
C1—H1A	0.9600	C11—C12	1.383 (5)
C1—H1B	0.9600	C11—H11	0.9300
C1—H1C	0.9600	C12—C13	1.374 (6)
C2—O1	1.367 (4)	C12—H12	0.9300
C2—C3	1.373 (5)	C13—N3	1.323 (5)
C2—C7	1.431 (4)	C13—H13	0.9300
C3—C4	1.397 (4)	C14—N3	1.332 (4)
C3—H3	0.9300	C14—H14	0.9300
C4—C5	1.359 (5)	C15—Sn1	2.092 (3)
C4—H4	0.9300	C15—H15A	0.9600
C5—C6	1.405 (4)	C15—H15B	0.9600
C5—H5	0.9300	C15—H15C	0.9600
C6—C7	1.406 (4)	C16—Sn1	2.098 (3)
C6—C8	1.430 (4)	C16—H16A	0.9600
C7—O2	1.327 (4)	C16—H16B	0.9600
C8—N2	1.287 (4)	C16—H16C	0.9600
C8—H8	0.9300	N1—N2	1.400 (3)
C9—O3	1.290 (3)	N2—Sn1	2.200 (3)
C9—N1	1.304 (4)	O2—Sn1	2.138 (2)
C9—C10	1.489 (4)	O3—Sn1	2.176 (2)
C10—C11	1.377 (4)		
			
O1—C1—H1A	109.5	C11—C12—H12	120.8
O1—C1—H1B	109.5	N3—C13—C12	124.6 (3)
H1A—C1—H1B	109.5	N3—C13—H13	117.7
O1—C1—H1C	109.5	C12—C13—H13	117.7
H1A—C1—H1C	109.5	N3—C14—C10	124.5 (3)
H1B—C1—H1C	109.5	N3—C14—H14	117.7
O1—C2—C3	123.7 (3)	C10—C14—H14	117.7
O1—C2—C7	115.5 (3)	Sn1—C15—H15A	109.5
C3—C2—C7	120.8 (3)	Sn1—C15—H15B	109.5
C2—C3—C4	121.1 (3)	H15A—C15—H15B	109.5
C2—C3—H3	119.4	Sn1—C15—H15C	109.5
C4—C3—H3	119.4	H15A—C15—H15C	109.5
C5—C4—C3	119.3 (3)	H15B—C15—H15C	109.5
C5—C4—H4	120.4	Sn1—C16—H16A	109.5
C3—C4—H4	120.4	Sn1—C16—H16B	109.5
C4—C5—C6	121.2 (3)	H16A—C16—H16B	109.5
C4—C5—H5	119.4	Sn1—C16—H16C	109.5
C6—C5—H5	119.4	H16A—C16—H16C	109.5
C5—C6—C7	120.8 (3)	H16B—C16—H16C	109.5
C5—C6—C8	115.1 (3)	C9—N1—N2	110.7 (2)
C7—C6—C8	124.2 (3)	C8—N2—N1	114.8 (2)
O2—C7—C6	124.5 (3)	C8—N2—Sn1	128.1 (2)
O2—C7—C2	118.7 (3)	N1—N2—Sn1	117.03 (18)
C6—C7—C2	116.8 (3)	C13—N3—C14	116.1 (3)
N2—C8—C6	127.9 (3)	C2—O1—C1	116.2 (3)
N2—C8—H8	116.0	C7—O2—Sn1	131.74 (19)
C6—C8—H8	116.0	C9—O3—Sn1	114.66 (19)
O3—C9—N1	125.6 (3)	C15—Sn1—C16	146.08 (14)
O3—C9—C10	117.4 (3)	C15—Sn1—O2	95.11 (11)
N1—C9—C10	117.0 (3)	C16—Sn1—O2	94.66 (12)
C11—C10—C14	117.5 (3)	C15—Sn1—O3	92.46 (12)
C11—C10—C9	123.8 (3)	C16—Sn1—O3	92.19 (12)
C14—C10—C9	118.7 (3)	O2—Sn1—O3	155.02 (9)
C10—C11—C12	119.0 (4)	C15—Sn1—N2	106.13 (11)
C10—C11—H11	120.5	C16—Sn1—N2	107.23 (11)
C12—C11—H11	120.5	O2—Sn1—N2	83.30 (9)
C13—C12—C11	118.3 (4)	O3—Sn1—N2	71.73 (9)
C13—C12—H12	120.8		
			
O1—C2—C3—C4	179.1 (3)	C6—C8—N2—N1	−179.5 (3)
C7—C2—C3—C4	−1.4 (5)	C6—C8—N2—Sn1	−0.4 (4)
C2—C3—C4—C5	0.2 (5)	C9—N1—N2—C8	−177.8 (3)
C3—C4—C5—C6	−0.3 (5)	C9—N1—N2—Sn1	3.0 (3)
C4—C5—C6—C7	1.5 (5)	C12—C13—N3—C14	−0.2 (6)
C4—C5—C6—C8	−178.7 (3)	C10—C14—N3—C13	−1.2 (5)
C5—C6—C7—O2	−179.7 (3)	C3—C2—O1—C1	−2.4 (5)
C8—C6—C7—O2	0.5 (5)	C7—C2—O1—C1	178.0 (4)
C5—C6—C7—C2	−2.5 (4)	C6—C7—O2—Sn1	−5.1 (4)
C8—C6—C7—C2	177.7 (3)	C2—C7—O2—Sn1	177.7 (2)
O1—C2—C7—O2	−0.5 (4)	N1—C9—O3—Sn1	−4.5 (4)
C3—C2—C7—O2	179.9 (3)	C10—C9—O3—Sn1	174.16 (19)
O1—C2—C7—C6	−177.9 (3)	C7—O2—Sn1—C15	110.8 (3)
C3—C2—C7—C6	2.4 (4)	C7—O2—Sn1—C16	−101.7 (3)
C5—C6—C8—N2	−177.5 (3)	C7—O2—Sn1—O3	3.7 (4)
C7—C6—C8—N2	2.3 (5)	C7—O2—Sn1—N2	5.1 (3)
O3—C9—C10—C11	177.3 (3)	C9—O3—Sn1—C15	−102.0 (2)
N1—C9—C10—C11	−3.9 (4)	C9—O3—Sn1—C16	111.6 (2)
O3—C9—C10—C14	−4.0 (4)	C9—O3—Sn1—O2	5.6 (4)
N1—C9—C10—C14	174.7 (3)	C9—O3—Sn1—N2	4.2 (2)
C14—C10—C11—C12	−0.1 (5)	C8—N2—Sn1—C15	−95.7 (3)
C9—C10—C11—C12	178.5 (3)	N1—N2—Sn1—C15	83.4 (2)
C10—C11—C12—C13	−1.1 (6)	C8—N2—Sn1—C16	90.5 (3)
C11—C12—C13—N3	1.3 (6)	N1—N2—Sn1—C16	−90.4 (2)
C11—C10—C14—N3	1.3 (5)	C8—N2—Sn1—O2	−2.3 (2)
C9—C10—C14—N3	−177.4 (3)	N1—N2—Sn1—O2	176.8 (2)
O3—C9—N1—N2	1.0 (4)	C8—N2—Sn1—O3	177.0 (3)
C10—C9—N1—N2	−177.6 (2)	N1—N2—Sn1—O3	−3.86 (19)
